# NEKfla: A Genetically Encoded Förster Resonance Energy Transfer Biosensor for Live-Cell Monitoring of Early NIMA-Related Kinase 7–NLR Family Pyrin Domain Containing 3 Inflammasome Signaling

**DOI:** 10.34133/bmr.0403

**Published:** 2026-09-07

**Authors:** Sanghyun Ahn, Jung-Soo Suh, Yoonkwan Jang, Gyuho Choi, Kiseok Han, Yerim Lee, Dahee Lee, Chanhui Song, Jeong-Min Go, Minji Kim, Jinyoung Lee, Jung-Hwan Lee, Hae-Won Kim, Hwayoung Yun, Tae-Jin Kim

**Affiliations:** ^1^ Department of Integrated Biological Science, College of Natural Sciences, Pusan National University, Busan 46241, Republic of Korea.; ^2^ Institute of Systems Biology, Pusan National University, Busan 46241, Republic of Korea.; ^3^ College of Pharmacy and Research Institute for Drug Development, Pusan National University, Busan 46241, Republic of Korea.; ^4^ Institute of Tissue Regeneration Engineering (ITREN), Dankook University, Cheonan 31116, Republic of Korea.; ^5^ Department of Biological Sciences, College of Natural Sciences, Pusan National University, Busan 46241, Republic of Korea.

## Abstract

The NLRP3 inflammasome is a key regulator of inflammatory responses and is increasingly recognized as an important mediator of pathological reactions to diverse chemical, biological, and microenvironmental stimuli. However, most currently available approaches primarily detect downstream or end-stage events, limiting real-time analysis of the early molecular processes that precede inflammasome assembly. Here, we report NEKfla, a genetically encoded Förster resonance energy transfer (FRET)-based biosensor designed to monitor early NEK7–NLRP3-associated signaling in living cells with single-cell spatiotemporal resolution. NEKfla was engineered using full-length NEK7 and NLRP3 linked to a ECFP–YPet FRET pair, and a ΔLRR control sensor was generated to assess interaction-dependent responses. In live-cell and in vitro analyses, NEKfla detected stimulus-dependent increases in FRET in response to lipopolysaccharide and nigericin, responded to pharmacological inhibition by MCC950 and licochalcone B, and visualized dynamic changes associated with NLRP3 oligomerization. The biosensor also functioned in multiple cellular contexts, including macrophage-like cells, and detected signaling changes earlier than a caspase-1 reporter. Together, these findings establish NEKfla as a live-cell platform for tracking proximal inflammasome activation events and support its potential utility in mechanistic studies and cell-based screening of materials or compounds that modulate early NLRP3 inflammasome signaling.

## Introduction

Inflammation is an essential host-defense response that protects tissues against infection and injury. However, persistent or dysregulated inflammatory signaling contributes to the development and progression of a broad range of human diseases [1]. Chronic inflammatory conditions, including inflammatory bowel disease, rheumatoid arthritis, Crohn’s disease, and encephalitic disorders, continue to impose a major clinical burden [2,3]. Accordingly, elucidating the molecular mechanisms that regulate inflammatory signaling remains critical for both mechanistic understanding and therapeutic intervention.

Among the key processes that drive inflammatory pathology, pyroptosis and inflammasome activation have emerged as central mechanisms [1,4]. Pyroptosis is an inflammatory form of programmed cell death that is closely coupled to the assembly of inflammasomes, supramolecular signaling complexes that promote caspase-1 activation and cytokine maturation [5]. Canonical inflammasome activation is generally understood as a 2-step process consisting of priming and activation. During priming, pathogen-associated molecular patterns (PAMPs), damage-associated molecular patterns (DAMPs), and cytokines induce the expression of inflammasome components and inflammatory cytokines [6,7]. During activation, pattern-recognition receptors assemble into oligomeric complexes, recruit apoptosis-associated speck-like protein (ASC), and nucleate ASC filaments that facilitate pro-caspase-1 recruitment and activation [8]. Activated caspase-1 subsequently promotes the maturation of pro-inflammatory cytokines and the cleavage of gasdermin D, resulting in membrane pore formation, release of inflammatory mediators, and amplification of immune responses [9]. When this process becomes excessive or unresolved, persistent pyroptotic signaling can contribute to chronic inflammation and tissue injury in diverse disease settings [10].

The inflammasome network comprises several classes of pattern-recognition receptors, among which the NLRP3 inflammasome has received particular attention because of its unusually broad responsiveness to distinct cellular stress signals [11]. In contrast to receptors that respond to more restricted inputs, NLRP3 can be activated by viral, bacterial, and fungal infection, as well as by sterile stimuli such as ion flux, lysosomal damage, and reactive oxygen species [12,13]. Because of this broad sensitivity, NLRP3 is widely recognized as a major inflammatory signaling hub and an important target in studies of inflammatory disease mechanisms and intervention [14].

Despite substantial progress in defining the molecular framework of inflammasome activation, approaches for monitoring NLRP3 signaling in living cells remain limited. Most currently used assays rely on endpoint or downstream readouts, such as the expression or activity of inflammasome-related proteins, ASC speck formation, or reporter systems centered on late signaling outputs [15,16]. Live-cell approaches based on fluorescent protein exchange (FPX) or bioluminescent resonance energy transfer (BRET) have expanded the experimental toolkit for inflammasome studies [17]. However, these reporters generally monitor caspase-1 activation, which occurs after inflammasome assembly and therefore provides only limited insight into the proximal molecular events that initiate aberrant NLRP3 activation [18]. This limitation has hindered high-resolution analysis of early inflammasome dynamics and constrained the development of cell-based platforms for evaluating early inflammatory signaling responses.

A pivotal proximal event in canonical NLRP3 inflammasome activation is the interaction between NLRP3 and NIMA-related kinase 7 (NEK7) [19,20]. NEK7 is a conserved serine/threonine kinase that contributes to cellular homeostasis, and its dysregulation has been linked to cell death and inflammatory processes [21]. In macrophages, NEK7 functions as an essential component of the NLRP3 inflammasome by directly regulating NLRP3 activity. Structural studies have shown that NEK7 binds the leucine-rich repeat (LRR) domain of NLRP3 and thereby promotes inflammasome assembly [22,23]. In addition, regions of NEK7 not directly engaged in NLRP3 binding contribute to oligomerization, which is required for productive inflammasome formation. Consistent with this model, NEK7 deficiency abolishes potassium efflux-induced NLRP3 inflammasome activation, including oligomer formation, ASC speck assembly, caspase-1 activation, and IL-1β release, without equivalently affecting NLRC4 or AIM2 inflammasome activation [20]. Together, these observations identify the NEK7–NLRP3 interface as an attractive target for monitoring early NLRP3 inflammasome signaling.

Here, we report NEKfla, a genetically encoded Förster resonance energy transfer (FRET)-based biosensor designed to monitor early NEK7–NLRP3-associated signaling in living cells. NEKfla was engineered to detect stimulus-dependent changes linked to the interaction between NEK7 and the NLRP3 LRR region with single-cell spatiotemporal resolution, and a control biosensor lacking the LRR domain (NEKfla ΔLRR) was generated to assess interaction-dependent responses. By enabling real-time visualization of signaling events that occur upstream of caspase-1 activation, this biosensor provides a useful platform for mechanistic studies of inflammasome initiation and for cell-based evaluation of compounds or materials that modulate early NLRP3 inflammasome activation.

## Materials and Methods

### Cell culture and reagents

HepG2, HEK293A, U87MG, and Raw 264.7 cells were obtained from the Korean Cell Line Bank (Republic of Korea). Cells were maintained in Dulbecco’s modified Eagle’s medium (DMEM; CM001-050, GenDEPOT, Katy, TX, USA) supplemented with 10% fetal bovine serum (FBS; WB0015, HyClone, Logan, UT, USA), 2 mM l-glutamine, 100 U ml^−1^ penicillin, and 100 μg ml^−1^ streptomycin (CA005, GenDEPOT) in T25 flasks (70025, SPL, Republic of Korea) at 37 °C in a humidified incubator with 5% CO_2_. Transient transfection of HepG2, HEK293A, and U87MG cells was performed using X-tremeGENE 360 Transfection Reagent (XTG360-RO, Roche, Switzerland) according to the manufacturer’s instructions. For Raw 264.7 cells, cells were first incubated with NATE (Nucleic Acid Transfection Enhancer) 1× (InvivoGen, San Diego, CA, USA) for 30 min and were then transfected with biosensor plasmids complexed with PEIpro transfection reagent (115-0015, Polyplus, France) at a 1:1 ratio of DNA (μg) to reagent (μl).

Nigericin (Nig; CS-5717) was purchased from ChemScene (Monmouth Junction, NJ, USA), lipopolysaccharide (LPS; L4391) from Sigma-Aldrich (St. Louis, MO, USA), MCC950 (S7809) from Selleckchem (Houston, TX, USA), licochalcone B (Lico B; HY-N0373) and *N*-formyl-Met-Leu-Phe (fMLP; HY-P0224) from MedChemExpress (Monmouth Junction, NJ, USA), WKYMVm (1800) and AC2-26 (1845) from Tocris (Bristol, UK), and dimethyl sulfoxide (DMSO; AC4002-050-00) from Biosesang (Seongnam, Republic of Korea).

### Generation of the FPR1-knockout U87MG cell line

U87MG cells were cotransfected with formyl peptide receptor 1 (FPR1) CRISPR/Cas9 KO Plasmid (h2) (sc-418173-KO-2, Santa Cruz Biotechnology, USA) and FPR1 HDR Plasmid (h2) (sc-418173-HDR-2, Santa Cruz Biotechnology, USA) using X-tremeGENE 360 according to the manufacturer’s protocol. At 72 h after transfection, cells were switched to fresh medium containing puromycin and subjected to antibiotic selection for 5 to 6 d. The resulting cell population was validated for loss of *FPR1* expression by reverse transcription polymerase chain reaction (RT-PCR), quantitative PCR (qPCR), and Western blotting before use in live-cell imaging experiments.

### DNA constructs and plasmids

The NEK7–NLRP3 interaction biosensor (NEKfla) was generated by inserting full-length human NEK7 and human NLRP3 cDNAs into the pCAGGS-6017nes vector, placing NEK7 between the N terminus of Turquoise-GL and the C terminus of YPet through an EV (Eevee; extension for enhanced visualization by evading extraFRET) linker. Turquoise-GL was subsequently replaced with enhanced cyan fluorescent protein (ECFP), and the final NEKfla construct was subcloned into pcDNA3.1 by inserting the NEK7–EV linker–NLRP3–ECFP fragment into the TAUCON plasmid through *Xho*I/*Xba*I digestion (Fig. S1). Full-length human NEK7 was amplified by PCR from pcDNA3-N-HA-NEK7 using the following primers: forward, 5​′-C​CCG​CTC​GAG​ATG​GAT​GAA​CAA​TCA​CAA​GGAATGC-3′; reverse, 5′-CATGGGTACCGGTGCTTGCGGTACATGC-3′. Full-length human NLRP3 was amplified from pEGFP-C2-NLRP3 using the following primers: forward, 5′-CATGTCCGGAATGAAGATGGCAAGCACCCGC-3′; reverse, 5​′-C​ATG​GCG​GCC​GCA​CCA​AGA​AGG​CTC​AAA​GACGAC-3′. The pCAGGS-6017nes vector was provided by M. Matsuda (Kyoto University) and is available from Addgene (#108655). The TAUCON construct was described previously [24].

Plasmids used in this study included pcDNA3-N-HA-NEK7, pEGFP-C2-NLRP3, the single-polypeptide FPX caspase-1 biosensor, FPR1-Tango, DsRed2-Mito-7, mCherry-ER-3, and mCherry-TGNP-N-10 (Addgene plasmids #75142, #73955, #60885, #66283, #55838, #55041, and #55145, respectively). The NEKfla ΔLRR construct was generated from NEKfla using the EZchange Site-Directed Mutagenesis Kit (EZ004S, Enzynomics, Republic of Korea) with the following primers: forward, 5′-TGGTGCGGCCGCATGGTG-3′; reverse, 5′-CTTTTCCTCCTCCTCTTCCTCCTTGGG-3′ (Fig. S2). Cloning and mutagenesis workflows were designed using SnapGene software (Fig. S3).

### In vitro spectrofluorometry

HEK293A cells were seeded in 70-mm culture dishes (TCD010070, JET BIOFIL, Guangzhou, China) and transfected with the NEKfla plasmid by calcium phosphate coprecipitation. At 36 h after transfection, cells were treated for 1 h with DMSO or 5 μM Nig. Cells were then lysed in Pierce radioimmunoprecipitation assay (RIPA) lysis and extraction buffer (89900, Thermo Fisher Scientific, USA), and clarified lysates from Nig-treated samples were divided into 3 aliquots and further incubated for 30 min at 37 °C with DMSO, 10 μM MCC950, or 20 μM Lico B. Lysates were transferred to white-bottom 96-well plates (30196, SPL, Republic of Korea), and fluorescence emission spectra from 460 to 560 nm were acquired using a CLARIOstar Plus microplate reader (BMG Labtech, Germany) with excitation at 433 nm.

### Colocalization analysis and FRET ratio quantification

Colocalization between NEKfla and subcellular organelle markers [DsRed2-Mito-7 for mitochondria, mCherry-ER-3 for endoplasmic reticulum, and mCherry-TGNP-N-10 for trans-Golgi network (TGN)] was analyzed using the JACoP (Just Another Colocalization Plugin) in Fiji/ImageJ (NIH, USA). Prior to analysis, background was subtracted from both channels using the rolling-ball algorithm (radius = 50 pixels), and regions of interest (ROIs) corresponding to individual cells were manually defined. Pearson’s correlation coefficient (PCC) was calculated using Costes’ automatic thresholding to ensure unbiased threshold selection. For organelle-specific FRET ratio quantification, binary masks were generated from the organelle marker channel using Otsu’s thresholding, and the FRET/ECFP ratio within each organelle-derived ROI and the cytosolic region was measured.

### Live-cell imaging and microscopy

For imaging experiments, transfected cells were replated onto coverglass-bottom dishes (200350, SPL, Republic of Korea) and serum-starved for 12 h in DMEM containing 0.5% FBS. Cells were then washed with phosphate-buffered saline (PBS) and transferred to CO_2_-independent medium (18045-088, Gibco, Waltham, MA, USA) supplemented with 0.5% FBS, 4 mM l-glutamine, 100 U ml^−1^ penicillin, and 100 μg ml^−1^ streptomycin. Imaging was performed on a Leica Dmi8 microscope equipped with a charge-coupled device camera (DFC450C, Leica, Germany) and a 100× HC PL FLUOTAR oil-immersion objective (numerical aperture, 1.32).

For imaging of NEKfla and NEKfla ΔLRR, the CYR71010 filter cube was used with excitation at 436/28 nm and emission at 473/22 nm with a 459-nm dichroic mirror for ECFP, and excitation at 436/28 nm and emission at 539/24 nm with a 459-nm dichroic mirror for FRET. YPet imaging used excitation at 506/21 nm and emission at 539/24 nm with a 523-nm dichroic mirror. For the single-polypeptide FPX caspase-1 biosensor, red fluorescence was acquired using excitation at 578/24 nm and emission at 641/78 nm with a 598-nm dichroic mirror (CYR71010 filter cube), whereas green fluorescence was acquired using excitation at 479/33 nm and emission at 539/24 nm with a 500-nm dichroic mirror (DFT51010 filter cube). Enhanced green fluorescent protein (EGFP) imaging used the DFT51010 cube, and mCherry or DsRed2 imaging used the CYR71010 cube. Images were acquired and processed using LAS X software (Leica, Germany), including background subtraction for all fluorescence channels.

### Quantification of oligomeric structures

To quantify changes in the number of NLRP3-associated puncta in NEKfla-expressing cells, cells were cultured on coverglass-bottom dishes, serum-starved for 12 h in DMEM containing 0.5% FBS, and transferred to CO_2_-independent medium before imaging. Baseline images were acquired before drug treatment. Cells were then treated in separate dishes with DMSO, Nig, or LPS for 3 h and re-imaged under identical microscope settings. Puncta were quantified from fluorescence images using ImageJ with the automatic nuclei counter plugin (UCSB Center for Bio-image Informatics).

### Fluorescence intensity and FRET image analysis

For fluorescence quantification, ROIs were drawn around the entire cell body of biosensor-expressing cells. Where indicated, cytosolic and punctate ROIs were analyzed separately. Background intensities were measured for the relevant channels, including red fluorescence, green fluorescence, YPet, ECFP, and FRET. Pixel-wise red/green ratio images for the FPX reporter were generated according to the following equation:

$$R_{\mathrm{red}/\mathrm{green}}\;=\;\frac{I_{\mathrm{red},\mathrm{ROI}}-I_{\mathrm{red},\mathrm{bg}}}{I_{\mathrm{green},\mathrm{ROI}}-I_{\mathrm{green},\mathrm{bg}}}$$ (1)

Pixel-wise FRET/ECFP ratio images for NEKfla were generated using the following equation:

$$R_{\mathrm{FRET}/\mathrm{ECFP}}\;=\;\frac{I_{\mathrm{FRET},\mathrm{ROI}}-I_{\mathrm{FRET},\mathrm{bg}}}{I_{\mathrm{ECFP},\mathrm{ROI}}-I_{\mathrm{ECFP},\mathrm{bg}}}$$ (2)

In both cases, *I* represents the measured signal intensity in the indicated channel. Ratio images were displayed in intensity-modulated mode, in which pixel brightness was determined by ECFP intensity and pixel color by the FRET/ECFP emission ratio. Quantitative analyses were performed using GraphPad Prism 9.0.

### RT-PCR and qPCR

Total RNA was isolated using TransZol Up reagent (TransGen Biotech, Beijing, China) according to the manufacturer’s instructions. cDNA synthesis was performed using the SmartGene Compact cDNA Synthesis Kit (SG-CDNAC100, SmartGene, Lausanne, Switzerland). RT-PCR was carried out using the Q5 Hot Start High-Fidelity PCR Kit (New England Biolabs, Ipswich, MA, USA). Primer sequences were as follows: *FPR1* forward, 5′-CCAAACCAGTGACACAGCTACC-3′; reverse, 5′-CAGCCTAACTCAAGGTGAGACG-3′; *GAPDH* forward, 5′-CGACCACTTTGTCAAGCTCA-3′; reverse, 5′-AGGGGTCTACATGGCAACTG-3′. PCR products were analyzed by gel electrophoresis and quantified with ImageJ.

Quantitative PCR was performed on a QuantStudio 1 Real-Time PCR System (Applied Biosystems, Thermo Fisher Scientific, USA) using ZV SY VKN 2× qPCR M.Mix (SYBR with Low ROX) (ZV-VK120-500, Zenvia, Brazil). Relative expression levels were calculated using the ΔΔ*C*_t_ method and normalized to GAPDH.

### Western blotting

U87MG and FPR1-knockout (KO) U87MG cells were lysed in Pierce RIPA lysis and extraction buffer (89900, Thermo Fisher Scientific, USA) supplemented with Halt Protease and Phosphatase Inhibitor Cocktail (78442, Thermo Fisher Scientific, USA). Protein concentrations were determined using the Pierce BCA Protein Assay Kit (23227, Thermo Fisher Scientific, USA). Samples were mixed with 4× NuPAGE LDS sample buffer (2885618, Invitrogen, USA) and 10× Bolt sample reducing agent (3139797, Invitrogen, USA), separated by 15% sodium dodecyl sulfate–polyacrylamide gel electrophoresis (SDS-PAGE), and transferred to Immobilon-P polyvinylidene difluoride membranes (IPNH00010, Merck Millipore, USA). Membranes were blocked in 5% skim milk prepared in TBS-T (ProNA 10× TBS-T, TLP-118.1, TransLab, Republic of Korea) for 1 h at room temperature.

Primary antibodies against FPR1 and β-actin were incubated overnight at 4 °C, followed by horseradish peroxidase-conjugated secondary antibodies for 1 h at room temperature. The following antibodies were used: anti-FPR1 (PA1-41398, Invitrogen, 1:1000), anti-β-actin (sc-47778, Santa Cruz Biotechnology, 1:200), m-IgGκ BP-HRP (mouse IgGκ binding protein conjugated to horseradish peroxidase; sc-516102, Santa Cruz Biotechnology, 1:10000), and mouse anti-rabbit IgG-HRP (sc-2357, Santa Cruz Biotechnology, 1:10000). Bands were visualized using SuperSignal West Pico PLUS chemiluminescent substrate (34580, Thermo Fisher Scientific, USA) and imaged with an iBright 1500 imaging system (A44114, Invitrogen, Carlsbad, CA, USA). Band intensities were quantified using ImageJ, and β-actin was used as a loading control.

### Statistical analysis

**Fig. 1.**
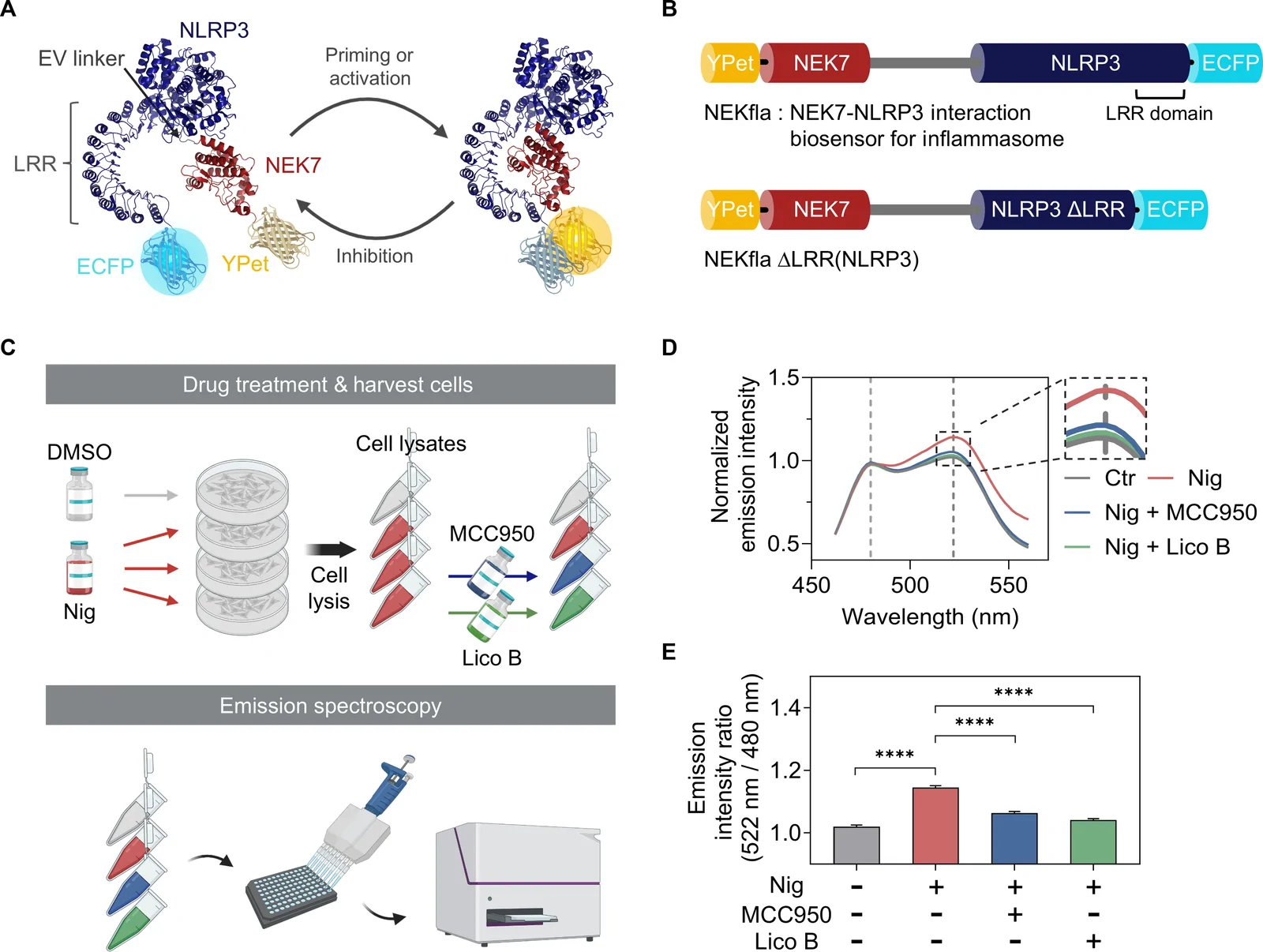
Design and in vitro characterization of the NEK7–NLRP3 FRET biosensor. (A) Schematic illustration of the proposed response mechanism of NEKfla. Increases in NEK7–NLRP3-associated signaling are expected to increase FRET, whereas inhibition of NLRP3 activity or disruption of NEK7–NLRP3 association is expected to reduce the FRET signal. Images were created using structural graphics for NLRP3–NEK7 (PDB 6NPY). (B) Domain organization of NEKfla and the control sensor NEKfla ΔLRR. NEKfla comprises YPet, full-length NEK7, an EV linker, full-length NLRP3, and ECFP. NEKfla ΔLRR lacks the LRR domain of NLRP3. (C) Schematic overview of the experimental workflow used for in vitro validation of NEKfla. All cells were transfected with NEKfla. Schematics were created with BioRender. (D) Normalized fluorescence emission spectra of NEKfla cell lysates excited at 433 nm after treatment with DMSO (Ctr), nigericin (Nig), Nig + MCC950, or Nig + licochalcone B (Lico B). Dashed lines indicate the emission maxima of ECFP (480 nm) and YPet (522 nm). (E) Quantification of the 522/480-nm emission ratio under the indicated conditions. Data are presented as mean ± SEM (*n* = 24 independently prepared cell lysates per condition; ****P < 0.0001, Student’s *t* tests).

Data analysis was performed using GraphPad Prism 9.0. Representative images and quantitative data were obtained from at least 3 independent experiments unless otherwise indicated, and *n* represents the number of individual cells analyzed. For the in vitro spectrofluorometric analysis shown in Fig. 1D and E, *n* represents the number of independently prepared cell lysates. Data are presented as mean ± SEM. For comparisons between 2 groups at a single time point or condition, statistical differences were assessed using unpaired 2-tailed Student’s *t* tests. For time-course data comparing 2 groups across multiple time points, 2-way analysis of variance (ANOVA) was used to evaluate the main effects of treatment/genotype and time. The specific statistical test applied to each figure is indicated in the corresponding figure legend. Differences were considered statistically significant at *P < 0.05, **P < 0.01, ***P < 0.001, and ****P < 0.0001.

The coefficient of variation [CV = (*σ*/μ) × 100; *σ*, standard deviation; *μ*, mean] and signal-to-noise ratio [SNR = (*μ*_signal_ − *μ*_background_)/*σ*_background_; *μ*_signal_ and *μ*_background_, mean FRET/ECFP ratios of treatment and control groups; *σ*_background_, standard deviation of control group] of the FRET/ECFP ratio were calculated to assess assay reproducibility, with values reported in the corresponding figure legends.

## Results

### Development and in vitro validation of a NEK7–NLRP3 FRET biosensor

To monitor an early step in canonical NLRP3 inflammasome activation, we designed a FRET-based biosensor to report NEK7–NLRP3-associated signaling. The biosensor, termed NEKfla, was constructed by linking full-length NEK7 and NLRP3 to a YPet/ECFP FRET pair through an EV linker (Fig. 1A and B). Because NEK7 acts upstream of inflammasome complex formation and is required for NLRP3 activation in response to multiple stimuli [20], we first examined whether NEK7 expression affected NLRP3 oligomerization in a reconstituted system. In HEK293A cells, which lack major endogenous inflammasome components such as NLRP3, ASC, and caspase-1 [25], coexpression of NEK7 increased NLRP3 oligomer formation relative to control conditions, and this effect was further enhanced by Nig treatment in a dose-dependent manner (Fig. S4). Consistent with the reported roles of MCC950 and Lico B in suppressing NLRP3 signaling [26,27], both compounds reduced NLRP3 oligomer formation under these conditions.

On the basis of structural studies showing close association between NEK7 and the LRR domain of NLRP3 during activation [23], we reasoned that activation-dependent binding would decrease the donor–acceptor distance and increase FRET efficiency. As a control, we generated NEKfla ΔLRR, in which the LRR domain of NLRP3 was deleted (Fig. 1B). In the resting state, or when upstream signaling is suppressed, the predicted donor–acceptor separation is expected to maintain a relatively low FRET state, characterized by higher ECFP emission at 480 nm and lower YPet emission at 522 nm upon excitation at 433 nm. By contrast, activation-associated NEK7 engagement is expected to increase the FRET/ECFP emission ratio.

To evaluate biosensor performance in vitro, cell lysates containing NEKfla were prepared from transfected HEK293A cells and subjected to fluorescence spectral analysis after pharmacological treatment (Fig. 1C). Nig increased the 522-nm emission peak relative to control conditions, whereas MCC950 and Lico B reduced this response. Quantification of the 522/480-nm emission ratio showed an approximately 1.14-fold increase in the Nig-treated group, whereas treatment with MCC950 or Lico B reduced the ratio to 0.93- and 0.91-fold of the Nig group, respectively (Fig. 1D and E). While MCC950 and Lico B effectively disrupt the NLRP3 activation and NLRP3–NEK7 interaction, they may not be able to completely dissociate the interaction, which remains stable during short in vitro incubation time, when compared to the control group. These data indicate that NEKfla reports treatment-dependent spectral changes consistent with early NEK7–NLRP3-associated signaling.

### Subcellular localization and functional dynamics of NEKfla

**Fig. 2.**
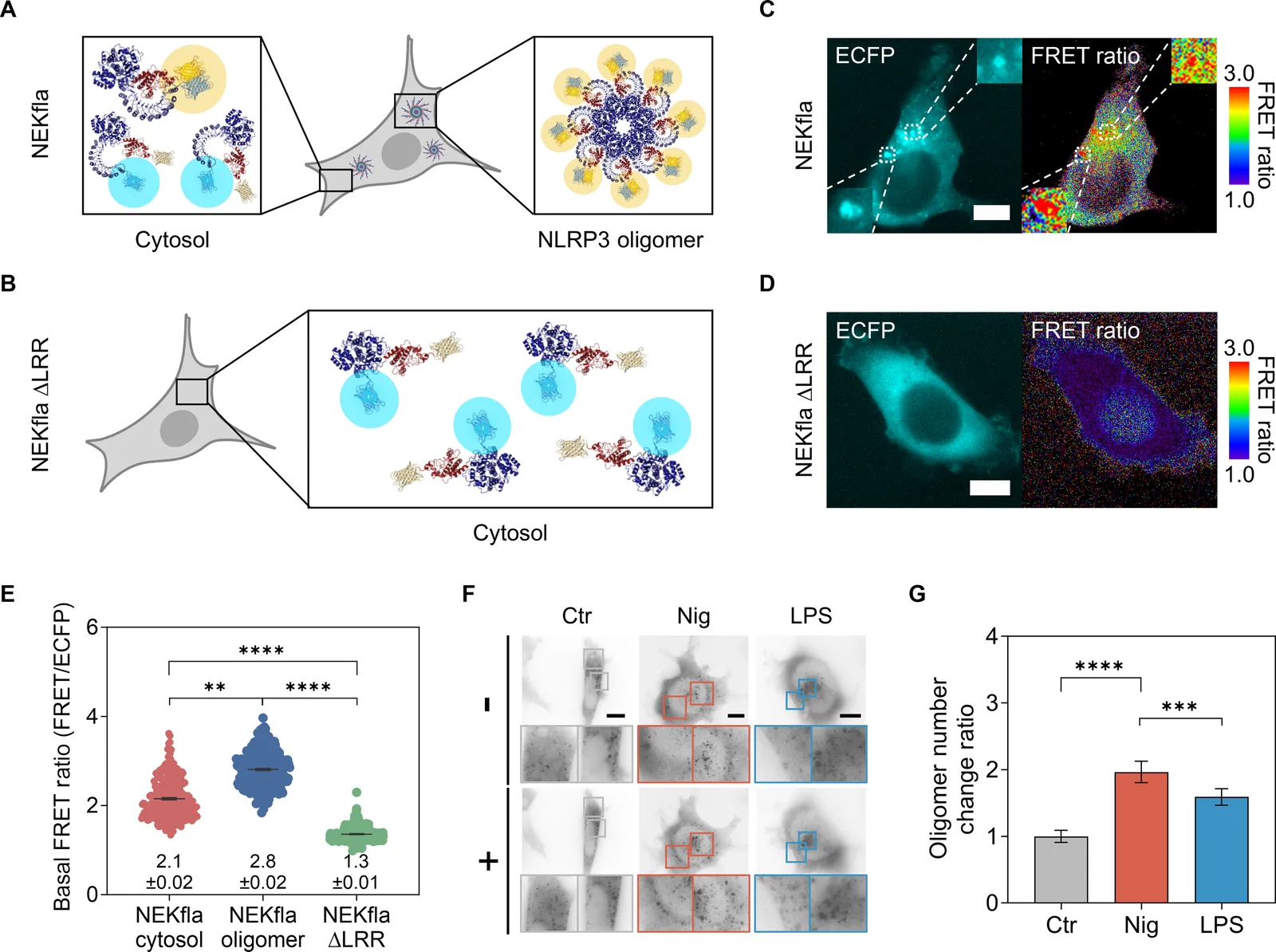
Subcellular distribution and puncta-associated NEKfla activity in HepG2 cells. (A and B) Schematic models illustrating the intracellular distribution of NEKfla (A) and NEKfla ΔLRR (B). Schematics were created with BioRender. (C and D) Representative fluorescence images of HepG2 cells expressing NEKfla or NEKfla ΔLRR, shown as donor-channel images (ECFP) and pseudocolored FRET/ECFP ratio images. Scale bars, 10 μm. (E) Basal FRET/ECFP ratios measured in cytosolic and punctate regions of interest (ROIs) in cells expressing NEKfla or NEKfla ΔLRR. Data are shown as scatterplots with mean ± SEM (*n* = 200; **P < 0.01, ****P < 0.0001, Student’s *t* tests). (F) Representative grayscale images (inverted ECFP − oligomer count analysis) of NEKfla-expressing HepG2 cells before and after treatment with DMSO, 5 μM Nig, or 1 μg ml^−1^ LPS. Insets highlight punctate regions. Scale bars, 10 μm. (G) Quantification of changes in puncta number under the indicated conditions. Data are presented as mean ± SEM (*n* = 8, 12, and 7 for DMSO, Nig, and LPS, respectively; ***P < 0.001, ****P < 0.0001, Student’s *t* tests).

To investigate the subcellular distribution of the biosensors, we performed live-cell fluorescence imaging in HepG2 cells expressing NEKfla or NEKfla ΔLRR, a cell line widely used in inflammasome-related studies (Fig. 2A to D) [28]. NEKfla was detected throughout the cytosol and also appeared in discrete punctate structures, whereas NEKfla ΔLRR showed a predominantly diffuse cytosolic pattern. In addition, the FRET/ECFP ratio of NEKfla was higher in punctate regions than in the surrounding cytosol, whereas NEKfla ΔLRR exhibited uniformly low ratios in both compartments (Fig. 2C and D). Because overexpression of foreign DNA can promote a priming-like cellular state and increase inflammasome-related signaling [29], the basal distribution and activity of NEKfla were interpreted cautiously. Under these conditions, the observed NEKfla pattern likely reflects both diffuse cytosolic localization and localized high-ratio puncta, whereas the ΔLRR control remains comparatively inactive (Fig. 2A and B).

Previous studies have shown that NEK7 and NLRP3 oligomerize before full inflammasome assembly [8,23]. In addition, receptor for activated protein C kinase 1 (RACK1), which is expressed in HepG2 cells, has been implicated in this oligomerization process [30]. On this basis, we considered the punctate structures observed in NEKfla-expressing cells to be consistent with NLRP3 oligomer-related regions (Fig. 2A and C). To further assess this possibility, we measured the emission ratio (FRET/ECFP) in 200 cells and separately analyzed oligomeric and cytosolic ROIs (Fig. 2E). Oligomeric ROIs in NEKfla-expressing cells showed an emission ratio approximately 2.2-fold higher than that of NEKfla ΔLRR, whereas cytosolic ROIs showed an approximately 1.6-fold higher ratio than the control sensor. These results indicate that NEKfla reports spatially distinct signaling states, with higher activity associated with punctate structures.

We next performed live-cell imaging of cells cotransfected with NEKfla and organelle markers for mitochondria, endoplasmic reticulum (ER), and the trans-Golgi network (TGN), each tagged with a red fluorescent protein. Under these conditions, quantitative colocalization analysis revealed that NEKfla puncta showed the highest spatial overlap with the TGN marker (Pearson’s *r* = 0.84) compared to mitochondrial (*r* = 0.19) or ER (*r* = 0.21) markers (Fig. S5). Because activated NLRP3 has been reported to relocalize to the TGN, where it can serve as a scaffold for NLRP3 oligomerization and ASC polymerization [31], this observation is consistent with the interpretation that NEKfla puncta are associated with activation-related NLRP3-rich structures. We then treated NEKfla-expressing HepG2 cells with Nig and LPS, a potent PAMP and a strong inducer of inflammasome priming [32]. Since 1 μg/ml LPS has been reported to be noncytotoxic and is commonly used in inflammatory cell models [33], this concentration was used throughout the experiments. Both treatments significantly increased the abundance of punctate regions relative to control cells (Fig. 2F and G), indicating that inflammatory stimulation promoted redistribution of NEKfla from a predominantly diffuse cytosolic pattern toward more localized punctate structures. Collectively, these findings support that NEKfla dynamically reports subcellular changes associated with early NLRP3 inflammasome signaling.

### Functional characterization and sensitivity validation of NEKfla

**Fig. 3.**
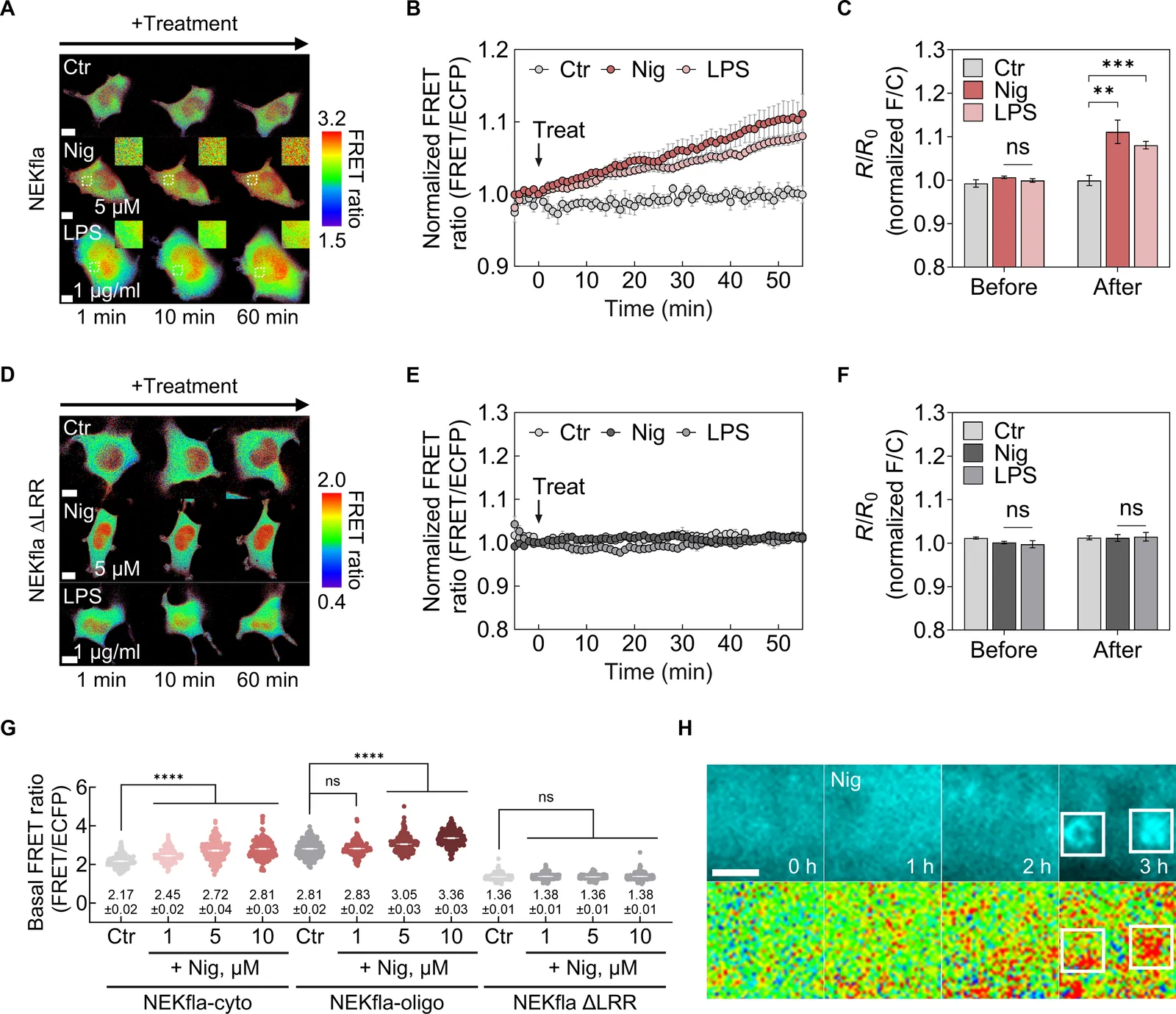
NEKfla reports stimulus-dependent inflammasome-associated signaling in HepG2 cells. (A and D) Representative time-lapse FRET/ECFP ratio images of HepG2 cells expressing NEKfla (A) or NEKfla ΔLRR (D) after treatment with DMSO (Ctr; 0.5% v/v), 5 μM Nig, or 1 μg ml^−1^ LPS. Warm colors indicate higher ratios. Scale bars, 10 μm. (B and E) Time courses of normalized FRET/ECFP ratios in cells expressing NEKfla (B) or NEKfla ΔLRR (E) under the indicated conditions. Data are presented as mean ± SEM. (C and F) Summary of normalized ratio changes before and after treatment in cells expressing NEKfla (C) or NEKfla ΔLRR (F). Data are presented as mean ± SEM (NEKfla: *n* = 5, 8, and 9; NEKfla ΔLRR: *n* = 5, 8, and 8 for DMSO, Nig, and LPS, respectively; **P < 0.01, ***P < 0.001, Student’s *t* tests). The coefficient of variation (CV) of NEKfla FRET ratio was 2.21% (5 μM Nig) and 2.24% (LPS), respectively. The signal-to-noise ratio (SNR) of NEKfla FRET ratio was 8.65 (5 μM Nig) and 6.13 (LPS), respectively. (G) FRET/ECFP ratios measured in cytosolic and punctate ROIs of HepG2 cells expressing NEKfla or NEKfla ΔLRR after 3-h treatment with 1, 5, or 10 μM Nig. Data are shown as scatterplots with mean ± SEM (*n* = 200; ****P < 0.0001, Student’s *t* tests). (H) Representative enlarged donor-channel and FRET/ECFP ratio images of NEKfla-expressing HepG2 cells treated with 10 μM Nig. Scale bars, 2 μm.

To evaluate the ability of NEKfla to report NEK7–NLRP3-associated signaling during inflammasome activation, we induced inflammatory signaling in HepG2 cells expressing the biosensor. As shown in Fig. 3A to C, the emission ratio (FRET/ECFP) of NEKfla gradually increased over 55 min after treatment with 1 μg/ml LPS or 5 μM Nig, reaching levels approximately 1.11- and 1.08-fold higher than those of DMSO-treated control cells, respectively. In addition, live-cell imaging performed with different concentrations of Nig (1 and 10 μM) revealed dose-dependent changes in the emission ratio, with increases of approximately 1.06-fold and 1.20-fold, respectively (Fig. S6). These results indicate that NEKfla is responsive to graded inflammatory stimulation in living cells.

Inflammasome activation is generally understood as a 2-step process consisting of priming and activation [14]. LPS acts primarily as a priming stimulus (step 1) of the NLRP3 inflammasome that drives transcriptional up-regulation of the inflammasome components and induces acute posttranslational modifications (PTMs) to facilitate the binding of NLRP3–NEK7. In contrast, Nig, as a potassium ionophore, induces robust intracellular K^+^ efflux, serving as a potent activation stimulus (step 2) that directly facilitates the physical interaction between NLRP3 and NEK7 and subsequent supramolecular assembly. In this context, the increase in NEKfla signal after Nig treatment is consistent with activation-associated enhancement of proximal NLRP3 signaling. Likewise, the moderate increase observed after LPS treatment may reflect priming-associated changes in NLRP3, including PTMs such as palmitoylation and phosphorylation, that facilitate subsequent NEK7 interaction [34,35]. Previous studies have also shown that LPS alone can increase NEK7–NLRP3 binding [36]. However, because LPS treatment without a subsequent activating stimulus does not typically induce full inflammasome hyperactivation, the difference between LPS- and Nig-induced responses in NEKfla likely reflects distinct signaling states rather than equivalent pathway output (Fig. 3C). To examine whether these FRET changes depended on the NLRP3 LRR region, we tested NEKfla ΔLRR under the same conditions (Fig. 3D to F and Fig. S6). In contrast to NEKfla, the control sensor showed little or no change in emission ratio relative to DMSO-treated cells.

To further assess the sensitivity of NEKfla, we analyzed the emission ratio (FRET/ECFP) in approximately 200 HepG2 cells expressing either NEKfla or NEKfla ΔLRR after 3-h treatment with 1, 5, or 10 μM Nig (Fig. 3G). Cytosolic and oligomeric ROIs were analyzed separately. Relative to NEKfla ΔLRR, NEKfla showed higher basal emission ratios in both cytosolic and oligomeric ROIs, and these ratios increased further in a dose-dependent manner after Nig treatment. Notably, increasing Nig concentrations elevated the cytosolic FRET ratio toward the basal level observed in oligomeric ROIs, suggesting a transition from diffuse cytosolic signaling to formation of localized high-ratio structures. This interpretation is consistent with the increase in NEKfla-positive puncta observed under the same conditions (Fig. 2F and G and Fig. S4). Time-lapse imaging further showed the emergence of high-ratio puncta in the cytosol during sustained stimulation with 10 μM Nig (Fig. 3H). Together, these results support that NEKfla dynamically reports signaling changes associated with early inflammasome activation and puncta formation.

We next examined whether NEKfla could detect suppression of early inflammasome-associated signaling. High-resolution live-cell imaging showed that treatment with 20 μM Lico B or 10 μM MCC950 gradually reduced the NEKfla emission ratio over 1 h, decreasing it to approximately 0.89-fold of the DMSO-treated control level (Fig. S7A to C). Under the same conditions, NEKfla ΔLRR remained largely unchanged (Fig. S7D to F). When cytosolic and oligomeric ROIs were analyzed after 3-h treatment, NEKfla ΔLRR again remained at basal levels, whereas NEKfla showed a reduction in oligomeric ROI signal toward the original cytosolic level (Fig. S7G). These findings are consistent with a decrease in activation-associated high-ratio puncta and support the utility of NEKfla for detecting pharmacological inhibition of proximal NLRP3 signaling.

**Fig. 4.**
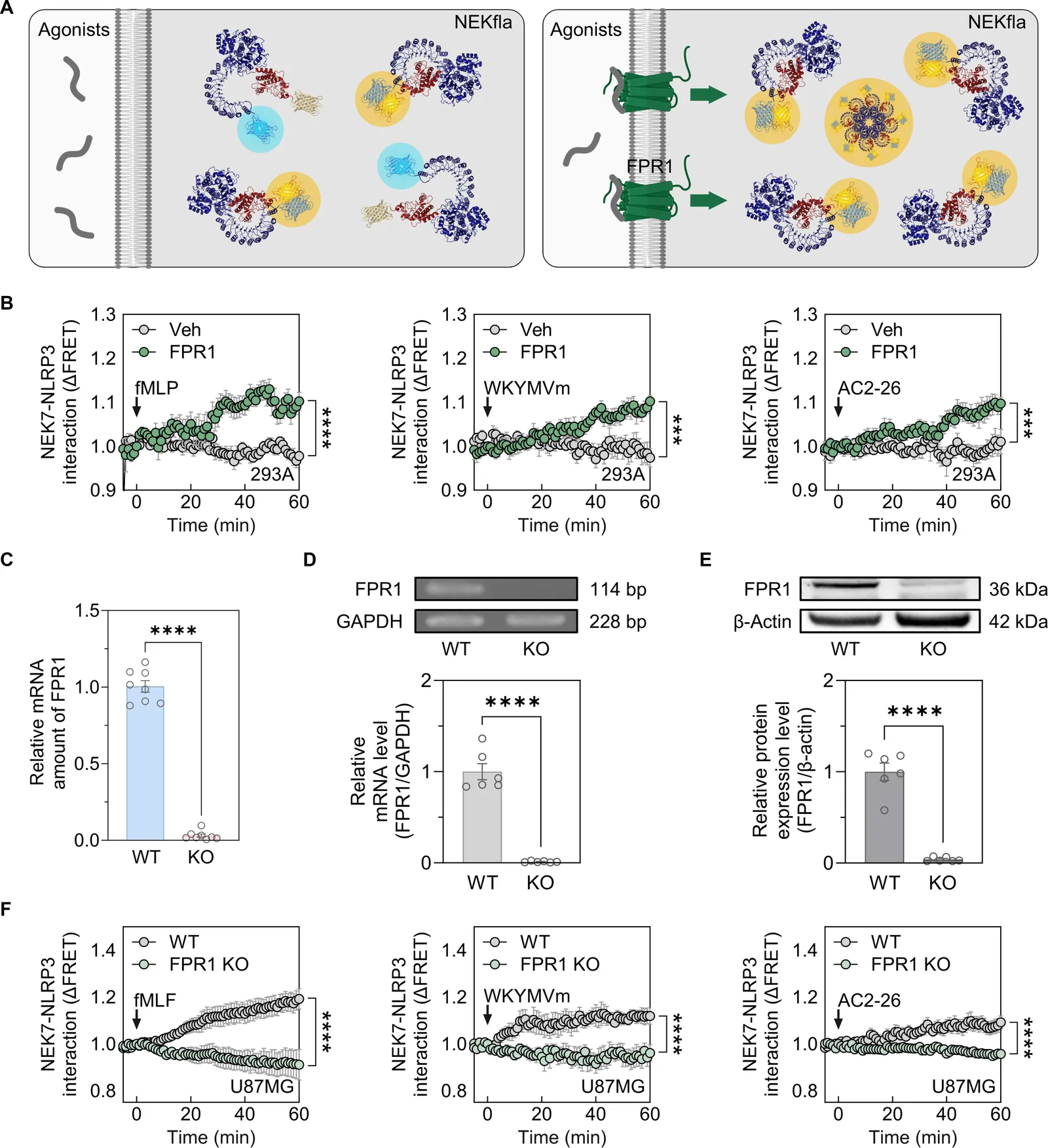
NEKfla responds to FPR1-associated immune stimulation. (A) Schematic illustration of the experimental design used to test NEKfla responses downstream of FPR1 activation. The schematic was created with BioRender. (B) Time courses of normalized FRET ratio changes (ΔFRET) in HEK293A cells expressing NEKfla alone (Veh) or coexpressing FPR1 and NEKfla after stimulation with 1 μM fMLP, 1 μM WKYMVm, or 1 μM AC2-26. Agonists were added at the indicated time points (arrows). Data are presented as mean ± SEM. Statistical significance was assessed by 2-way ANOVA, which revealed a significant interaction between FPR1 expression and time [fMLP: *F*(65,786) = 2.124, ****P < 0.0001; WKYMVm: *F*(65,726) = 3.239, ***P < 0.001; AC2-26: *F*(65,594) = 1.958, ***P < 0.001]. (C) Relative *FPR1* mRNA levels in wild-type (WT) and FPR1-knockout (KO) U87MG cells measured by qPCR and normalized to *GAPDH*. Data are presented as mean ± SEM (*n* = 8 per group; ****P < 0.0001, Student’s *t* tests). (D) Representative RT-PCR gel image (top) and quantification (bottom) of *FPR1* transcripts in WT and FPR1-KO U87MG cells. GAPDH was used as a loading control. Data are presented as mean ± SEM (*n* = 6 per group; ****P < 0.0001, Student’s *t* tests). (E) Representative Western blot (top) and densitometric analysis (bottom) of FPR1 protein levels in WT and FPR1-KO U87MG cells. β-Actin was used as a loading control. Data are presented as mean ± SEM (*n* = 6 per group; ****P < 0.0001, Student’s *t* tests). (F) Time courses of normalized FRET ratio changes (ΔFRET) in WT and FPR1-KO U87MG cells after stimulation with 1 μM fMLP, 1 μM WKYMVm, or 1 μM AC2-26. Agonists were added at the indicated time points (arrows). Data are presented as mean ± SEM. Statistical significance was assessed by 2-way ANOVA. The interaction between genotype (WT or FPR1-KO) and time was highly significant [fMLP: *F*(65,1254) = 3.412, ****P < 0.0001; WKYMVm: *F*(65,660) = 1.397, ****P < 0.0001; AC2-26: *F*(65,1300) = 4.312, ****P < 0.0001].

To further assess whether NEKfla can detect NLRP3 inflammasome signaling initiated by distinct upstream pathways, we examined its response to FPR1-mediated stimulation. FPR1 is a G protein-coupled receptor that has been reported to activate the NLRP3 inflammasome through Gαi-dependent downstream signaling following ligand engagement [37]. To test whether FPR1 activation influences the NEKfla signal, HEK293A cells, which do not endogenously express FPR1, were compared with cells coexpressing FPR1 and NEKfla (Fig. 4A). Cells expressing NEKfla alone (Veh) showed little or no change in FRET ratio after stimulation with fMLP, WKYMVm, or AC2-26. In contrast, cells coexpressing FPR1 and NEKfla exhibited an approximately 10% increase in FRET ratio after agonist treatment (Fig. 4B), indicating that FPR1-associated signaling can enhance NEK7–NLRP3-associated activity.

To validate this observation in a more physiologically relevant setting, we extended the analysis to U87MG cells, which endogenously express FPR1 and are widely used as an in vitro model for FPR1-related studies [38], together with an FPR1-KO derivative. Successful disruption of FPR1 expression in the KO cell line was confirmed at both the transcript and protein levels (Fig. 4C to E). In live-cell imaging experiments, FPR1-KO attenuated agonist-induced increases in the NEKfla signal relative to wild-type cells (Fig. 4F). Collectively, these findings indicate that NEKfla can report early inflammasome-associated signaling across multiple upstream inflammatory contexts and support its utility as a live-cell platform for monitoring proximal NLRP3 pathway activation.

### Early detection of NEK7–NLRP3 interaction prior to caspase-1 activation using NEKfla

The interaction between NEK7 and NLRP3 represents an early step in canonical inflammasome activation and precedes downstream events including NLRP3 oligomerization, recruitment of ASC and caspase-1, inflammasome assembly, and subsequent activation of caspase-1 and gasdermin D [19,20,39]. In contrast, many currently used approaches for detecting inflammasome activity primarily monitor later stages of the pathway, such as oligomer or ASC speck formation by fluorescence microscopy or caspase-1 activation using reporter-based assays [16,17]. Among these, the single-polypeptide FPX biosensor for caspase-1 is based on dimerization-dependent fluorescent protein (ddFP) technology, in which fluorescence output changes upon caspase-1-dependent processing of the reporter. Because this system reports a downstream enzymatic event, it provides a useful comparator for evaluating whether NEKfla can detect earlier signaling changes.

**Fig. 5.**
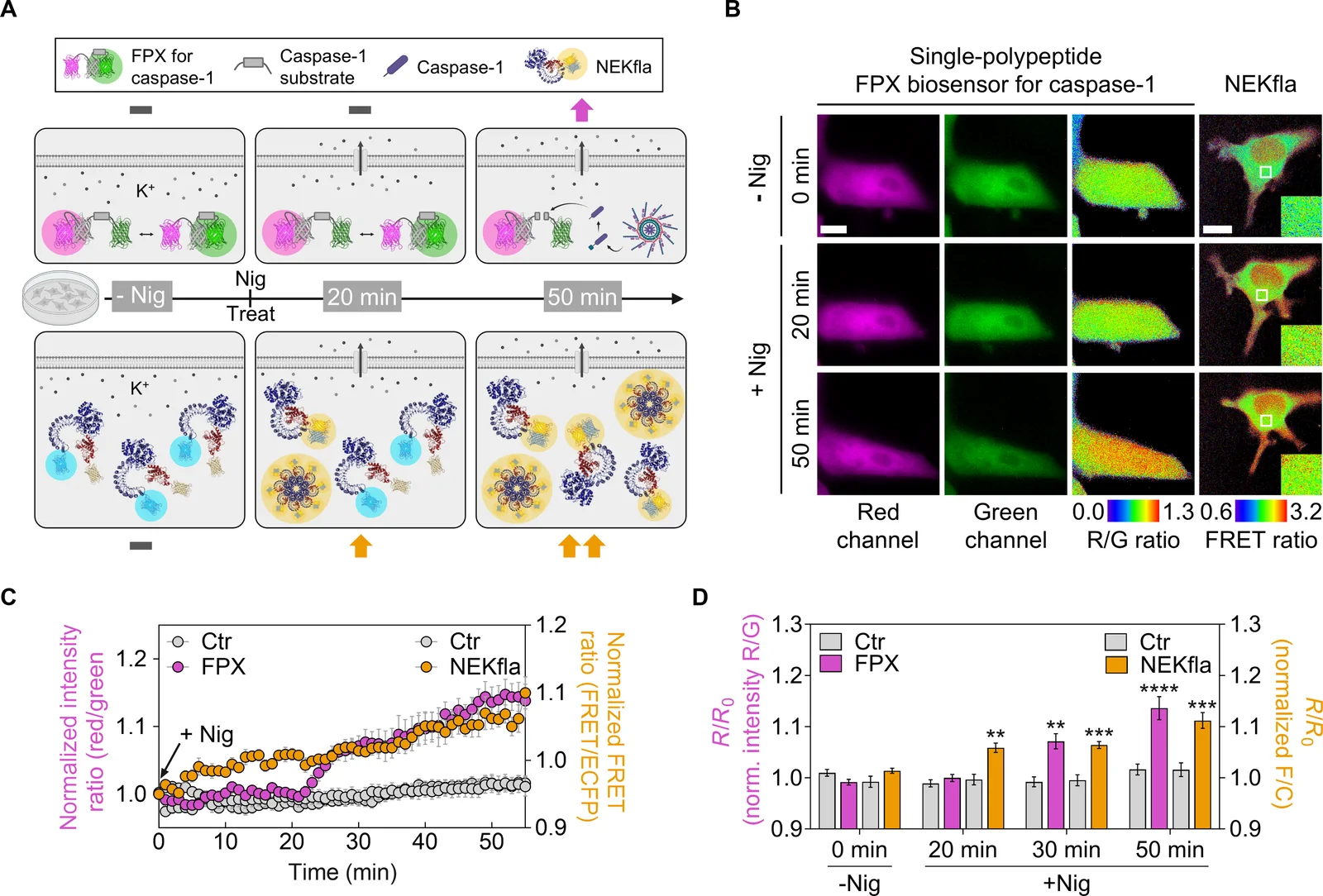
NEKfla detects proximal inflammasome-associated signaling earlier than a caspase-1 reporter. (A) Schematic comparison of NEKfla and the single-polypeptide FPX caspase-1 reporter. The schematic was created with BioRender. (B) Representative time-lapse images (fluorescence and R/G ratio) of the FPX reporter and corresponding FRET/ECFP ratio images of NEKfla-expressing HepG2 cells after Nig treatment. In merged FPX images, white and purple indicate higher and lower caspase-1 reporter activity, respectively. In ratio images, warm and cool colors indicate higher and lower NEK7–NLRP3-associated signaling, respectively. Scale bars, 10 μm. (C) Time courses of normalized red/green ratios for the FPX reporter (left *y* axis) and normalized FRET/ECFP ratios for NEKfla (right *y* axis) in HepG2 cells treated with DMSO or Nig. Data are presented as mean ± SEM (*n* = 17, 10, 16, and 6, respectively). (D) Summary of normalized signal changes at 20, 30, and 50 min after treatment for the FPX reporter and NEKfla. Data are presented as mean ± SEM (**P < 0.01, ***P < 0.001, ****P < 0.0001, Student’s *t* tests).

To determine whether NEKfla responds more rapidly than the single-polypeptide FPX biosensor, we compared the temporal dynamics of the 2 reporters by live-cell imaging for 1 h following Nig treatment (Fig. 5A to C). Both biosensors showed stimulus-dependent ratio changes relative to control cells, but their response kinetics differed. The FRET/ECFP ratio of NEKfla began to increase shortly after Nig treatment, whereas the red/green intensity ratio of the FPX biosensor increased only after an approximately 20-min delay (Fig. 5A and B). These observations indicate that NEKfla detects a proximal signaling event that occurs earlier than downstream caspase-1 activation in this cell-based system.

Quantitative analysis further supported this interpretation. The red/green ratio of the FPX biosensor remained near baseline during the initial 20 min and reached an approximately 1.13-fold increase at 50 min. In contrast, the FRET/ECFP ratio of NEKfla showed a significant increase relative to control by 20 min and reached an approximately 1.10-fold increase at 50 min (Fig. 5C and D). Together, these results support that NEKfla reports early NEK7–NLRP3-associated signaling before a detectable response from the downstream caspase-1 reporter and therefore provides a useful live-cell tool for monitoring proximal stages of NLRP3 inflammasome activation.

### NEKfla enables investigation of inflammasome-associated signaling in immune cells

**Fig. 6.**
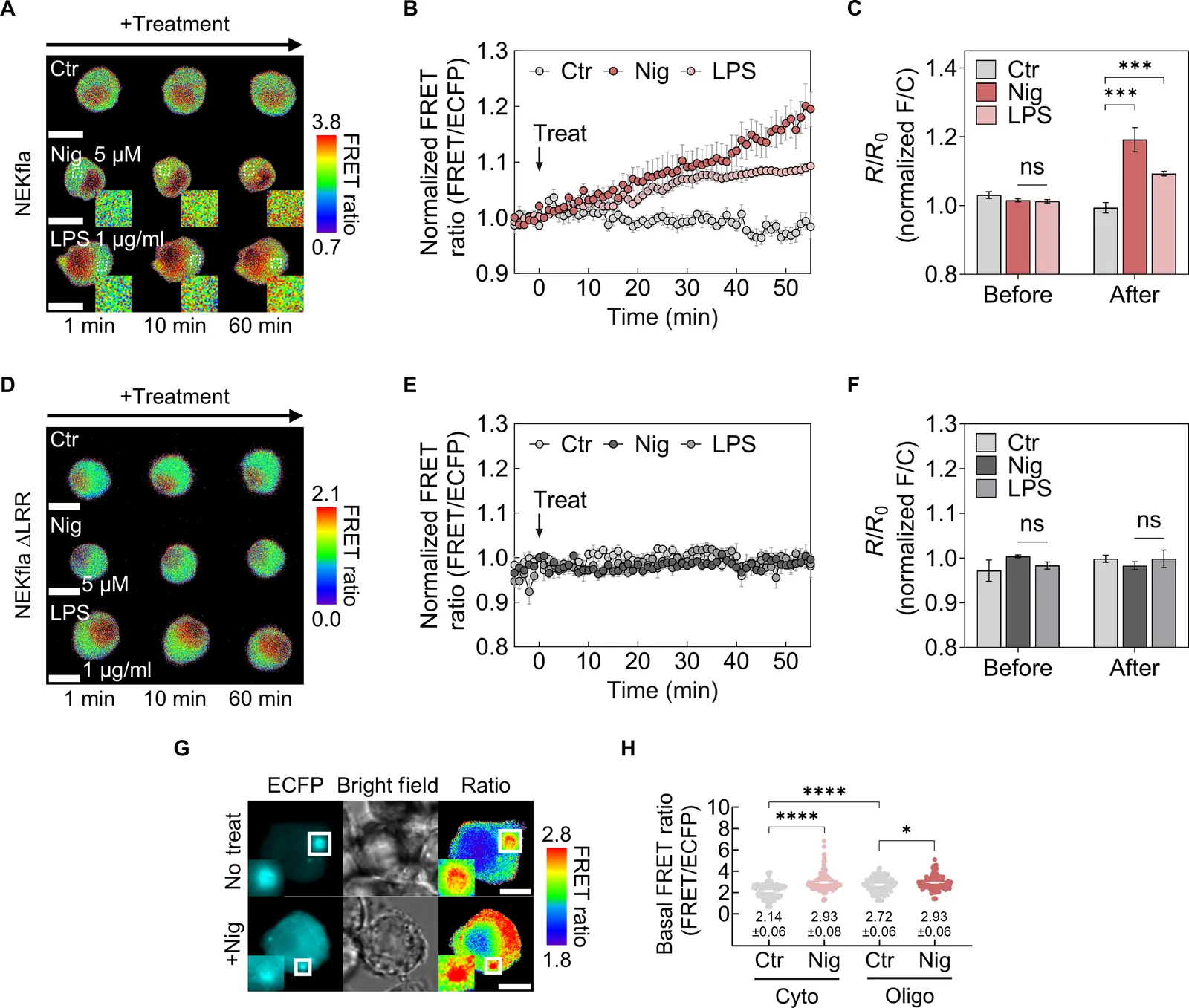
NEKfla monitors inflammasome-associated signaling in Raw 264.7 macrophage-like cells. (A and D) Representative time-lapse FRET/ECFP ratio images of Raw 264.7 cells expressing NEKfla (A) or NEKfla ΔLRR (D) after treatment with DMSO (Ctr; 0.5% v/v), 5 μM Nig, or 1 μg ml^−1^ LPS. Warm colors indicate higher ratios. Scale bars, 10 μm. (B and E) Time courses of normalized FRET/ECFP ratios in cells expressing NEKfla (B) or NEKfla ΔLRR (E) under the indicated conditions. Data are presented as mean ± SEM. (C and F) Summary of normalized ratio changes before and after treatment in cells expressing NEKfla (C) or NEKfla ΔLRR (F). Data are presented as mean ± SEM (NEKfla: *n* = 6, 9, and 6; NEKfla ΔLRR: *n* = 8, 9, and 9 for DMSO, Nig, and LPS, respectively; ***P < 0.001, Student’s *t* tests). The coefficient of variation (CV) of NEKfla FRET ratio was 4.54% (5 μM Nig) and 1.48% (LPS), respectively. The SNR of NEKfla FRET ratio was 9.23 (5 μM Nig) and 4.62 (LPS), respectively. (G) Representative donor-channel, bright-field, and FRET/ECFP ratio images of Raw 264.7 cells expressing NEKfla before and after Nig treatment. Scale bars, 10 μm. (H) FRET/ECFP ratios measured in cytosolic and punctate ROIs of Raw 264.7 cells expressing NEKfla before and after 3 h treatment with 5 μM Nig. Data are shown as scatterplots with mean ± SEM (*n* = 200; *P < 0.05, ****P < 0.0001, Student’s *t* tests).

To assess the applicability of NEKfla in immune-cell-related contexts, we examined its performance in Raw 264.7 macrophage-like cells, which play central roles in inflammatory responses. Cells were pretreated with NATE, transfected with either NEKfla or NEKfla ΔLRR, and subjected to live-cell imaging under the same conditions used in the previous experiments (Fig. 6 and Fig. S8). Following treatment with 1 μg/ml LPS or 5 μM Nig, the emission ratio (FRET/ECFP) of NEKfla progressively increased over 60 min (Fig. 6A and B). Relative to DMSO-treated control cells, the signal increased by approximately 1.19-fold after LPS treatment and 1.09-fold after Nig treatment (Fig. 6C). These findings indicate that NEKfla responds to inflammatory stimulation in macrophage-like cells.

To determine whether these responses depended on the NLRP3 LRR region, we tested NEKfla ΔLRR under the same conditions. In contrast to NEKfla, the control sensor showed little or no change in emission ratio relative to control cells across the analyzed regions (Fig. 6D to F). We further analyzed 200 NEKfla-expressing Raw 264.7 cells and quantified the emission ratio in cytosolic and oligomeric ROIs (Fig. 6G and H). Because Raw 264.7 cells express RACK1 [40], a factor associated with NLRP3 oligomer-related signaling, the higher FRET/ECFP ratio observed in punctate regions relative to the cytosol is consistent with a cellular context permissive for localized inflammasome-associated signaling. In addition, 3-h treatment with 5 μM Nig significantly increased the emission ratio in both cytosolic and oligomeric ROIs of NEKfla.

We next examined whether NEKfla could detect pharmacological suppression of proximal inflammasome-associated signaling in Raw 264.7 cells. Live-cell imaging showed that treatment with 20 μM Lico B or 10 μM MCC950 gradually reduced the emission ratio of NEKfla over 1 h relative to control cells (Fig. S8A to C). Under the same conditions, NEKfla ΔLRR remained largely unchanged (Fig. S8D to F). Collectively, these results indicate that NEKfla can report stimulus-dependent and inhibitor-sensitive signaling changes in macrophage-like cells, supporting its utility as a live-cell biosensor for monitoring early inflammasome-associated signaling in immune-cell-related settings.

## Discussion

Inflammation is essential for host defense and tissue homeostasis, but its dysregulation contributes to the pathogenesis of a broad range of diseases, including cardiovascular disorders, diabetes, neurodegenerative diseases, and cancer [41,42]. Among the pathways that drive inflammatory pathology, the NLRP3 inflammasome has emerged as a central signaling platform because it senses diverse stimuli, including extracellular adenosine triphosphate (ATP), nucleic acids, and ion flux, and transduces these inputs into downstream inflammatory responses [14]. Despite its importance, however, the proximal molecular events that initiate aberrant NLRP3 activation remain incompletely understood. Current models indicate that NEK7 selectively binds to NLRP3 during activation, an interaction that is uniquely required for the canonical NLRP3 pathway but is dispensable for other inflammasomes such as NLRC4 and AIM2 [20]. This NEK7–NLRP3 engagement promotes oligomerization and formation of a supramolecular complex that ultimately enables ASC-dependent caspase-1 activation. On the basis of this early signaling step, we developed the FRET-based biosensor NEKfla to monitor NEK7–NLRP3-associated signaling during the initial phase of NLRP3 inflammasome activation [43].

Our data demonstrate that NEKfla provides a live-cell readout of early inflammasome-associated signaling. In HepG2 cells, the biosensor detected stimulus-dependent changes in FRET in response to LPS and Nig, whereas the ΔLRR control showed little or no corresponding response. In addition, NEKfla activity decreased upon treatment with MCC950 and Lico B, supporting the interpretation that the observed signal is associated with proximal NLRP3 signaling. These findings indicate that NEKfla can sensitively visualize and quantify signaling changes linked to NEK7–NLRP3 association and NLRP3 oligomer-related dynamics. Importantly, comparison with the caspase-1 reporter further showed that NEKfla responds earlier than a downstream inflammasome readout, highlighting its utility for monitoring signaling events that precede terminal pathway outputs.

An important feature of NEKfla is its ability to report signaling changes during the transition from priming to activation. The moderate increase in FRET observed following LPS treatment is consistent with the concept that priming is not merely transcriptional, but is also accompanied by posttranslational licensing events that facilitate subsequent NLRP3 activation. By contrast, the stronger response to Nig is compatible with progression to a more activated state. In this regard, NEKfla may provide a useful experimental platform for distinguishing early permissive signaling from later inflammasome assembly events, which are more commonly assessed by ASC speck formation or caspase-1 activation.

At the same time, several considerations should temper interpretation of the biosensor signal. NEKfla is an engineered reporter based on full-length NEK7 and NLRP3 and therefore may not fully reproduce endogenous stoichiometry or kinetics. In addition, because biosensor overexpression itself may create a partially primed background state, the basal cytosolic activity and punctate structures observed in some cells should be interpreted cautiously. For this reason, NEKfla is best viewed as a reporter of NEK7–NLRP3-associated signaling states rather than as a strict one-to-one surrogate for endogenous complex formation under all conditions. Likewise, although our data support a close relationship between NEKfla puncta and NLRP3 oligomer-related structures, the formation of the NEK7–NLRP3 complex alone is unlikely to be sufficient for full inflammasome signaling, because productive NLRP3 oligomerization requires additional conformational regulation mediated by factors such as RACK1 [23,30].

The applicability of NEKfla across distinct cellular contexts further supports its utility. In addition to HepG2 cells, the biosensor responded to FPR1-mediated stimulation and functioned in Raw 264.7 macrophage-like cells. These results suggest that NEKfla can detect upstream inflammatory inputs that converge on the NLRP3 pathway. However, Raw 264.7 cells should still be interpreted with caution in the context of downstream inflammasome biology, because they are not optimal for fully resolving ASC-dependent signaling events [44]. Nevertheless, the elevated cytosolic FRET signal observed in Raw 264.7 cells may reflect a broader inflammatory signaling context associated with macrophage behavior, as previous studies have linked RACK1 to cell migration and invasion [45]. This observation raises the possibility that NEK7–NLRP3-associated signaling may also be dynamically coupled to macrophage activation-related cellular states beyond canonical endpoint inflammasome outputs.

From a translational perspective, NEKfla may be useful for evaluating early inflammasome signaling in response to inflammatory modulators and candidate therapeutic interventions. Chronic brain inflammation associated with amyloid-β deposition in Alzheimer’s disease is one example in which the NLRP3 inflammasome has been implicated as a pathogenic factor [46,47]. Likewise, excessive NLRP3 activation has been linked to SARS-CoV-2 infection and cytokine storm responses [48]. Because many currently used anti-inflammatory strategies target broad downstream responses, there remains strong interest in tools that can monitor earlier and potentially more selective regulatory nodes. In this context, NEKfla provides a potentially useful platform for studying compounds that modulate NEK7–NLRP3-associated signaling and for evaluating upstream regulatory mechanisms, including PTMs that contribute to NLRP3 activation. Overall, NEKfla helps bridge an important methodological gap between static biochemical assays and downstream functional reporters. By enabling real-time visualization of proximal NEK7–NLRP3-associated signaling in living cells, this biosensor should facilitate mechanistic studies of inflammasome initiation and support future screening efforts aimed at identifying modulators of early NLRP3 inflammasome activation.

## Conclusion

In summary, we developed NEKfla, a FRET-based biosensor that enables live-cell monitoring of early NEK7–NLRP3-associated inflammasome signaling. NEKfla responded to canonical NLRP3 stimuli, distinguished interaction-dependent responses from those of the ΔLRR control sensor, and detected signaling changes earlier than a caspase-1 reporter. The biosensor also functioned across multiple cellular contexts, including macrophage-like cells and FPR1-associated stimulation models. These findings establish NEKfla as a useful live-cell platform for investigating proximal events in NLRP3 inflammasome activation. With further validation in endogenous and application-relevant systems, NEKfla may support mechanistic studies and screening approaches aimed at identifying compounds that modulate early inflammasome-associated signaling.

## Data Availability

The data that support the findings of this study are available from the corresponding authors upon reasonable request.
