# Supplementary material for: NEKfla: A Genetically Encoded Förster Resonance Energy Transfer Biosensor for Live-Cell Monitoring of Early NIMA-Related Kinase 7–NLR Family Pyrin Domain Containing 3 Inflammasome Signaling
Source: Biomater Res. 2026 Sep 7;30:0403. doi: 10.34133/bmr.0403 (PMC13547735; doi:10.34133/bmr.0403)
Supplement: Supplementary file 1 [file bmr.0403.f1.docx]

**Supplementary Materials**

**NEKfla: A Genetically Encoded Förster Resonance Energy Transfer Biosensor for Live-Cell Monitoring of Early NIMA-related kinase 7–NLR family pyrin domain containing 3 Inflammasome Signaling**

Sanghyun Ahn^1^, Jung-Soo Suh^1,2^, Yoonkwan Jang^1^, Gyuho Choi^1^, Kiseok Han^1^, Yerim Lee^1^, Daehee Lee^1^, Chanhui Song^1^, Jeong-Min Go^1^, Minji Kim^1^, Jinyoung Lee^3^, Jung-Hwan Lee^4^, Hae-Won Kim^4^, Hwayoung Yun^3*^, and Tae-Jin Kim^1,2,5*^

^1^Department of Integrated Biological Science, College of Natural Sciences, Pusan National University, Busan 46241, Republic of Korea, ^2^ Institute of Systems Biology, Pusan National University, Busan 46241, Republic of Korea, ^3^College of Pharmacy and Research Institute for Drug Development, Pusan National University, Busan 46241, Republic of Korea, ^4^Institute of Tissue Regeneration Engineering (ITREN), Dankook University, Cheonan, 31116, Republic of Korea, ^5^Department of Biological Sciences, College of Natural Sciences, Pusan National University, Busan 46241, Republic of Korea

*Address correspondence to: [hyun@pusan.ac.kr](mailto:hyun@pusan.ac.kr) (H.Y.); [tjkim77@pusan.ac.kr](mailto:tjkim77@pusan.ac.kr) (T.-J.K.)

**Figures. S1-S8**

**SUPPLEMENTRARY MATERIALS**

**Figure. S1.** Map of NEK7-NLRP3 Interaction Biosensor for Inflammasome (NEKfla) Expression Plasmid. Schematic representation of the NEK7-NLRP3 interaction biosensor (NEKfla) expression plasmid, detailing the insertion sites and the arrangement of key components necessary for the expression of the biosensor in cells.

**Figure. S2.** Map of NEK7-NLRP3 Interaction Biosensor for Inflammasome (NEKfla) ΔLRR Expression Plasmid. Schematic representation of the NEK7-NLRP3 interaction biosensor (NEKfla) ΔLRR expression plasmid, highlighting the deletion of the LRR domain from NLRP3. This map details the insertion sites and arrangement of key components necessary for the expression of the ΔLRR mutant biosensor in cells.

**Figure. S3.** Sequence Alignment of NEK7-NLRP3 Interaction Biosensor for Inflammasome (NEKfla) and Its Deletion Mutants (NEKfla ΔLRR). Sequence alignment of the NEKfla and its deletion mutant NEKfla ΔLRR prepared through site-directed mutagenesis. Dashes denote deletions in the sequence.

**Figure. S4.** EGFP Images of NLRP3-EGFP in Live HEK293A Cells Co-transfected with pcDNA3-NEK7 or pcDNA3.1. EGFP images of NLRP3-EGFP in live HEK293A cells co-transfected with pcDNA3-NEK7 (red line) or pcDNA3.1 (blue line) treated with (A) 1 μM, 5 μM, and 10 μM Nigericin (Nig), and DMSO (Ctr, 0.5% v/v). (B) 20 μM Licochalcone B (Lico B), 10 μM MCC950, and DMSO (Ctr, 0.5% v/v). Green indicates NLRP3-EGFP, with images provided before (-) and after (+) drug treatment in one cell. Scale bar = 10 μm.

**Figure. S5.** Subcellular Localization of NEKfla in HepG2 Cells. (A) ECFP, DsRed2, mCherry, merged, and bright field images of NEKfla in HepG2 cells co-transfected with DsRed2-mito-7, mCherry-ER-3, or mCherry-TGNP-N-10. Blue indicates NEKfla, and red indicates organelle markers (mitochondria (top row), ER (middle row), or TGN (bottom row)). The two colors in each row are merged to show co-localization. Scale bar = 10 μm. (B) Quantification of colocalization between NEKfla puncta and organelle markers using Pearson's correlation coefficient (r), calculated with the JACoP plugin in Fiji. Data are presented as mean ± SEM (n = 15, respectively). (C) Basal FRET/ECFP ratios measured in cytosolic regions and organelle-defined ROIs (mitochondria, ER, TGN) in cells expressing NEKfla. Data are shown as scatter plots with mean ± SEM (n = 150; ****P < 0.0001, Student’s t-tests).

**Figure. S6.** Time-Lapse Analysis of NEKfla and NEKfla ΔLRR in Live HepG2 Cells. (A and D) Time-lapse images of the emission ratio (FRET/ECFP) for NEKfla (A) and NEKfla ΔLRR (D) were acquired in live HepG2 cells exposed to DMSO (Ctr; 0.5% v/v), 1 and 10 μM Nigericin (Nig). Warm and cool colors correspond to high and low NEK7–NLRP3 interaction levels, respectively (scale bar = 10 μm). (B) Time-course graphs of normalized FRET/ECFP emission ratios in NEKfla-expressing HepG2 cells treated with DMSO (0.5% v/v), 1, and 10 μM Nig (light gray, pink, and red; n = 9, 6, and 8, respectively). All error bars indicate SEM. (C) Mean normalized emission ratios of NEKfla (FRET/ECFP) before and after treatment. All error bars indicate SEM (light gray, pink, and red; n = 9, 6, and 8, respectively, **: p < 0.01, ****: p < 0.0001, Student’s t-test). The coefficient of variation (CV) of NEKfla FRET ratio was 3.82% (1 μM Nig) and 3.73% (10 μM Nig), respectively. The signal-to-noise ratio (SNR) of NEKfla FRET ratio was 4.76 (1 μM Nig) and 15.52 (10 μM Nig), respectively. (E) Time-course changes in normalized FRET/ECFP emission ratios for NEKfla ΔLRR in HepG2 cells treated with DMSO (0.5% v/v), 1, and 10 μM Nig (light gray, gray, and dark gray; n = 6, 8, and 8, respectively). All error bars indicate SEM. (F) Mean normalized emission ratios of NEKfla ΔLRR (FRET/ECFP) before and after treatment. All error bars indicate SEM (light gray, gray, and dark gray; n = 6, 8, and 8, respectively).

**Figure. S7.** NEKfla Response to NLRP3 Activity Inhibition and NEK7-NLRP3 Interaction Blockade. (A and D) Time-lapse images of the emission ratio (FRET/ECFP) for NEKfla (A) and NEKfla ΔLRR (D) were acquired in live HepG2 cells exposed to DMSO (Ctr; 0.5% v/v), 20 μM Lico B, and 10 μM MCC950. Warm and cool colors correspond to high and low NEK7–NLRP3 interaction levels, respectively (scale bar = 10 μm). (B) Time-course graphs of normalized emission ratios (FRET/ECFP) in NEKfla-expressing HepG2 cells treated with DMSO (0.5% v/v), 20 μM Lico B, and 10 μM MCC950 (light gray, blue, and light blue; n = 5, 7, and 8, respectively). All error bars indicate SEM. (C) Mean normalized emission ratios of NEKfla (FRET/ECFP) before and after treatment. All error bars indicate SEM (light gray, blue, and light blue; n = 5, 7, and 8, respectively, *: p < 0.05, **: p < 0.01, Student’s t-test). The coefficient of variation (CV) of NEKfla FRET ratio was 4.42% (Lico B) and 5.02% (MCC950), respectively. The signal-to-noise ratio (SNR) of NEKfla FRET ratio was 6.14 (Lico B) and 6.23 (MCC950), respectively. (E) Time-course changes in normalized emission ratios (FRET/ECFP) for NEKfla ΔLRR in HepG2 cells treated with DMSO (0.5% v/v), 20 μM Lico B, and 10 μM MCC950 (light gray, dark gray, and gray; n = 6 for each condition). All error bars indicate SEM. (F) Mean normalized emission ratios of NEKfla ΔLRR (FRET/ECFP) before and after treatment. All error bars indicate SEM (light gray, dark gray, and gray; n = 6 for each condition). (G) Basal emission ratios (FRET/ECFP) of NEKfla-expressing and NEKfla ΔLRR-expressing HepG2 cells. NEKfla ROIs were separated into cytosolic and oligomeric regions. Cells were then treated with 20 μM Lico B and 10 μM MCC950 for 3 h. Values are shown in a scatter plot with the mean±SEM value indicated by a line. The mean and SEM values are shown below the dot (n = 200, ****: p < 0.0001, Student’s t-test).

**Figure. S8.** NEKfla Application in Raw264.7 Cells: Monitoring Inflammasome Inhibition. (A and D) Time-lapse images of the FRET/ECFP emission ratio for NEKfla (A) and NEKfla ΔLRR (D) were acquired in live Raw264.7 cells exposed to DMSO (Ctr; 0.5% v/v), 20 μM Licochalcone B (Lico B), and 10 μM MCC950. Warm and cool colors correspond to high and low NEK7–NLRP3 interaction levels, respectively (scale bar = 10 μm). (B) Time-course graphs of normalized FRET/ECFP emission ratios in NEKfla-expressing Raw264.7 cells treated with DMSO (0.5% v/v), 20 μM Lico B, and 10 μM MCC950 (light gray, blue, and light blue; n = 6, 10, and 7, respectively). All error bars indicate SEM. (C) Mean normalized emission ratios of NEKfla (FRET/ECFP) before and after treatment. All error bars indicate SEM (light gray, blue, and light blue; n = 6, 10, and 7, respectively, ***: p < 0.001, ****: p < 0.0001, Student’s t-test). The coefficient of variation (CV) of NEKfla FRET ratio was 4.81% (Lico B) and 5.44% (MCC950), respectively. The signal-to-noise ratio (SNR) of NEKfla FRET ratio was 10.73 (Lico B) and 6.55 (MCC950), respectively. (E) Time-course changes in normalized FRET/ECFP emission ratios for NEKfla ΔLRR in Raw264.7 cells treated with DMSO (0.5% v/v), 20 μM Lico B, and 10 μM MCC950 (light gray, dark gray, and gray; n = 8, 9, and 11, respectively). All error bars indicate SEM. (F) Mean normalized emission ratios of NEKfla ΔLRR (FRET/ECFP) before and after treatment. All error bars indicate SEM (light gray, dark gray, and gray; n = 8, 9, and 11, respectively).
